# Supplementary material for: Modeling glioblastoma heterogeneity as a dynamic network of cell states
Source: Mol Syst Biol. 2021 Sep 16;17(9):e10105. doi: 10.15252/msb.202010105 (PMC8444284; doi:10.15252/msb.202010105)
Supplement: Supplementary file 6 — Source Data for Figure 5 [file MSB-17-e10105-s004.zip › Figure5A_sourcedata/GSEA_3017/nosigdb_stateB.GseaPreranked.1622795021725/RICHARDS_NOWAKOWSKI_SCIENCE_2017_VRG_UPREG.html]

Details for gene set RICHARDS\_NOWAKOWSKI\_SCIENCE\_2017\_VRG\_UPREG[GSEA]

|  || Dataset | state43017 |
| Phenotype | NoPhenotypeAvailable |
| Upregulated in class | na\_neg |
| GeneSet | RICHARDS\_NOWAKOWSKI\_SCIENCE\_2017\_VRG\_UPREG |
| Enrichment Score (ES) | -0.22548574 |
| Normalized Enrichment Score (NES) | -1.9778254 |
| Nominal p-value | 0.0 |
| FDR q-value | 0.014112773 |
| FWER p-Value | 0.048 |
Table: GSEA Results Summary

  

Fig 1: Enrichment plot: RICHARDS\_NOWAKOWSKI\_SCIENCE\_2017\_VRG\_UPREG      
 Profile of the Running ES Score & Positions of GeneSet Members on the Rank Ordered List

  

| PROBE | GENE SYMBOL | GENE\_TITLE | RANK IN GENE LIST | RANK METRIC SCORE | RUNNING ES | CORE ENRICHMENT || 1 | TAGLN2 |  |  | 61 | 0.604 | -0.0598 | No |
| 2 | GFAP |  |  | 132 | 0.487 | -0.1378 | No |
| 3 | CYR61 |  |  | 145 | 0.465 | -0.1337 | No |
| 4 | BARD1 |  |  | 189 | 0.427 | -0.1758 | No |
| 5 | SYNE2 |  |  | 203 | 0.413 | -0.1756 | No |
| 6 | PDPN |  |  | 205 | 0.410 | -0.1582 | No |
| 7 | ANXA2 |  |  | 211 | 0.408 | -0.1468 | No |
| 8 | AXL |  |  | 213 | 0.407 | -0.1296 | No |
| 9 | GPC4 |  |  | 217 | 0.404 | -0.1154 | No |
| 10 | SLC1A3 |  |  | 237 | 0.388 | -0.1249 | No |
| 11 | PPP1CB |  |  | 246 | 0.385 | -0.1187 | No |
| 12 | JADE1 |  |  | 251 | 0.378 | -0.1072 | No |
| 13 | ZFP36L2 |  |  | 279 | 0.358 | -0.1295 | No |
| 14 | DDAH1 |  |  | 300 | 0.346 | -0.1423 | No |
| 15 | DOK5 |  |  | 302 | 0.346 | -0.1279 | No |
| 16 | AKAP12 |  |  | 320 | 0.339 | -0.1368 | No |
| 17 | EMP2 |  |  | 333 | 0.333 | -0.1387 | No |
| 18 | NES |  |  | 334 | 0.332 | -0.1236 | No |
| 19 | FSTL1 |  |  | 362 | 0.321 | -0.1476 | No |
| 20 | EFNB2 |  |  | 374 | 0.317 | -0.1488 | No |
| 21 | MID1 |  |  | 375 | 0.317 | -0.1343 | No |
| 22 | SLC35F1 |  |  | 386 | 0.312 | -0.1344 | No |
| 23 | TTYH3 |  |  | 396 | 0.309 | -0.1331 | No |
| 24 | EZR |  |  | 403 | 0.307 | -0.1277 | No |
| 25 | MEIS2 |  |  | 405 | 0.307 | -0.1151 | No |
| 26 | FABP7 |  |  | 414 | 0.302 | -0.1127 | No |
| 27 | TMEM47 |  |  | 437 | 0.295 | -0.1308 | No |
| 28 | PHIP |  |  | 440 | 0.295 | -0.1202 | No |
| 29 | SYNE1 |  |  | 458 | 0.291 | -0.1312 | No |
| 30 | HSPA4L |  |  | 461 | 0.290 | -0.1208 | No |
| 31 | TTYH1 |  |  | 469 | 0.287 | -0.1177 | No |
| 32 | ACTN1 |  |  | 475 | 0.286 | -0.1118 | No |
| 33 | MOXD1 |  |  | 489 | 0.283 | -0.1175 | No |
| 34 | IFITM3 |  |  | 512 | 0.279 | -0.1363 | No |
| 35 | YBX3 |  |  | 519 | 0.278 | -0.1322 | No |
| 36 | CNN3 |  |  | 527 | 0.276 | -0.1296 | No |
| 37 | PDGFC |  |  | 532 | 0.275 | -0.1227 | No |
| 38 | DKK3 |  |  | 549 | 0.273 | -0.1332 | No |
| 39 | YAP1 |  |  | 560 | 0.270 | -0.1351 | No |
| 40 | RAI14 |  |  | 572 | 0.269 | -0.1386 | No |
| 41 | GNG12 |  |  | 587 | 0.267 | -0.1465 | No |
| 42 | PAX6 |  |  | 617 | 0.261 | -0.1761 | No |
| 43 | HEG1 |  |  | 639 | 0.257 | -0.1944 | No |
| 44 | TSPAN5 |  |  | 646 | 0.256 | -0.1913 | No |
| 45 | RDX |  |  | 658 | 0.255 | -0.1954 | No |
| 46 | SGCE |  |  | 680 | 0.250 | -0.2140 | Yes |
| 47 | HMGCR |  |  | 683 | -0.257 | -0.2052 | Yes |
| 48 | SCD |  |  | 688 | -0.263 | -0.1989 | Yes |
| 49 | MSMO1 |  |  | 691 | -0.267 | -0.1895 | Yes |
| 50 | VIM |  |  | 692 | -0.267 | -0.1773 | Yes |
| 51 | CA12 |  |  | 698 | -0.274 | -0.1720 | Yes |
| 52 | ID4 |  |  | 708 | -0.285 | -0.1718 | Yes |
| 53 | MYO10 |  |  | 710 | -0.305 | -0.1593 | Yes |
| 54 | ZEB1 |  |  | 717 | -0.317 | -0.1534 | Yes |
| 55 | FGFBP3 |  |  | 721 | -0.334 | -0.1424 | Yes |
| 56 | HSPB1 |  |  | 730 | -0.378 | -0.1366 | Yes |
| 57 | IDI1 |  |  | 734 | -0.397 | -0.1227 | Yes |
| 58 | SQLE |  |  | 735 | -0.405 | -0.1042 | Yes |
| 59 | HMGCS1 |  |  | 739 | -0.418 | -0.0894 | Yes |
| 60 | DBI |  |  | 740 | -0.422 | -0.0701 | Yes |
| 61 | PPA1 |  |  | 742 | -0.424 | -0.0521 | Yes |
| 62 | FABP5 |  |  | 759 | -1.641 | 0.0000 | Yes |
Table: GSEA details [plain text format]

  

Fig 2: RICHARDS\_NOWAKOWSKI\_SCIENCE\_2017\_VRG\_UPREG: Random ES distribution      
 Gene set null distribution of ES for **RICHARDS\_NOWAKOWSKI\_SCIENCE\_2017\_VRG\_UPREG**
